# On the influence of social norms on individual achievement goals

**DOI:** 10.1111/bjep.12756

**Published:** 2025-03-01

**Authors:** Sophie Bossert, Martin Daumiller, Stefan Janke, Markus Dresel, Oliver Dickhäuser

**Affiliations:** ^1^ Department of Psychology University of Mannheim Mannheim Germany; ^2^ Department of Psychology University of Augsburg Augsburg Germany

**Keywords:** achievement goals, contextual influence, peer influence, social norms, student motivation

## Abstract

**Background:**

Individual achievement goals are influenced by the learning context, such as the classroom. In this social space, social norms emerge and shape motivation and behaviour. Classroom goal structures reflect injunctive norms (what is considered acceptable) and influence individual achievement goals. The role that descriptive norms (what others typically do or think) play in individual achievement goals is unclear. We propose that peer achievement goals reflect descriptive norms and additionally influence individual achievement goals.

**Aims:**

We aim to better understand contextual influences on individual student motivation by applying a social norms framework to study changes in individual achievement goals and acknowledge the role of peers.

**Sample and Methods:**

We used longitudinal data from 4189 students from 169 classes at two time points after the transition to secondary school.

**Results:**

We calculated multilevel models to predict changes in individual mastery‐, performance‐approach, and performance‐avoidance goals. As Level‐2 predictors, class‐level classroom goal structures represented injunctive norms, while peer achievement goals represented descriptive norms. Individual achievement goals and individual‐level classroom goal structures were added on Level 1. Class‐level classroom goal structures related to changes in individual achievement goals only if peer achievement goals were not added. If added on the classroom level, peer achievement goals remained as a single Level‐2 predictor of changes in individual achievement goals.

**Conclusion:**

We demonstrated the key role that descriptive norms (reflected by peer achievement goals) play in individual achievement goals. The role of injunctive norms needs to be investigated further to enhance our understanding of how social norms shape individual student motivation.

## INTRODUCTION

Individual student motivation is highly relevant for educational psychologists (Koenka, 2020). It drives how students select, initiate, sustain, and direct behaviour and influences various student outcomes (Schunk & Mullen, 2012; Urhahne & Wijnia, 2023). Important antecedents of individual learning motivation lie in the context in which students learn (Roeser et al., 2009). A salient context is the classroom, where students interact with classmates and teachers, forming a social space. In this social space, shared rules of behaviour are formed and can influence behaviour. These social norms entail (1) expectations about what is appropriate or acceptable in a social group (injunctive norms) and (2) how others think or behave in a certain situation (descriptive norms) (Cialdini et al., 1990). Previous research has not fully addressed social norms in reasoning about contextual factors influencing individual achievement motivation. We aim to provide new knowledge on this issue by applying a social norms framework to contextual influences on individual achievement goals.

Achievement goals are well‐established as motivational factors in learning and influence various student outcomes (Elliot et al., 1999; Payne et al., 2007; Senko & Dawson, 2017). A better understanding of their antecedents is thus of great interest. In recent research, personal and contextual factors have been established (Butera et al., 2024; Elliot & Church, 1997). Applying a social norms framework, inferences about the role of injunctive norms can be drawn from research on classroom goal structures which, in our view, reflect this type of norm as they describe ‘messages in the learning environment (e.g., the classroom or school) that make certain goals salient’ (Urdan & Schoenfelder, 2006, p. 334). The role of descriptive norms established by peers in the classroom for individual achievement goals has been explored less, even though from a theoretical and empirical point of view, they can be assumed to be significant. Theoretically, peer achievement goals can be interpreted as descriptive social norms and may shape individual achievement goals. Empirically, peers emerged as the primary source of normative influence during adolescence (Brown, 2004).

We apply social norms to our reasoning on contextual influences on individual achievement goals and argue that injunctive and descriptive norms should be considered. In doing so, we explicitly address the importance of peers in the classroom. Our framework offers a novel approach, emphasizing how student motivation is shaped by their social reality.

### Social norms shape individual behaviour and motivation

Social norms are rules or expectations formed within a social group through interpersonal interactions and can be explicitly communicated or understood by observing others (Cialdini et al., 1990; Cialdini & Trost, 1998). They are seen as one of the essential drivers of individual behaviour (e.g., Legros & Cislaghi, 2020; Sherif, 1936) and serve as informal guidelines for what is considered acceptable or appropriate in different social situations. Individuals follow social norms for social reasons such as to gain approval or to avoid being punished in the respective social group, but they also use them as an information source about the most effective behaviour (Cialdini et al., 1991; Cialdini & Goldstein, 2004; Steinel et al., 2010).

There are two distinct categories of social norms (Cialdini et al., 1990, 1991; Reno et al., 1993). *Descriptive norms* refer to what is typically done by most people in a setting and reflect the prevalence of a behaviour. *Injunctive norms* refer to what others consider acceptable or approve of. Both descriptive and injunctive norms have been demonstrated to significantly impact individual behaviour and motivation across a wide range of fields (e.g., health, pro‐environmental behaviour, marketing; see Legros & Cislaghi, 2020 for an overview).

When studying social norms, it is important to bear in mind on which level they are represented. On the group level, norms can be understood as ‘prevailing codes of conduct that either prescribe or proscribe behaviors that members of a group can enact’ (Lapinski & Rimal, 2005, p. 129). Descriptive and injunctive norms thus describe the behaviour and attitudes belonging to members of a relevant reference group, conveying a strong sense of norms as a contextual factor. On the individual level, norms reflect individual perceptions about what others do or think one should do.

We propose that social norms as a contextual factor are useful to explain changes in individual achievement motivation and behaviour in the classroom. This is particularly plausible when it comes to goals that characterize the aims of achievement motivation. Personal goals are inherently anchored in personal values and beliefs but have also been shown to be affected by demands of the learning context. We consider social norms to be an important factor that helps the individual to comprehend which goals are deemed beneficial or seen as less desirable. A social norms approach is valuable not only in incorporating previously considered influences but also in identifying potential additional factors for individual student motivation that may have been previously overlooked.

### Individual achievement goals

Achievement Goals are understood as reasons or purposes for achievement‐related outcomes (Elliot & McGregor, 2001) and impact emotions, cognitions, and behaviour (Payne et al., 2007; Senko & Dawson, 2017). Since the early years of achievement goal research, two main types of goals have been distinguished: mastery goals and performance goals. Individuals with mastery goals strive to improve their knowledge or skills and to understand tasks. Individuals with performance goals strive to demonstrate competence and to perform better than others. Several models introducing new types of goals have been proposed, yet with no clear consensus on which additions are necessary. However, a further differentiation of performance goals according to an approach/avoidance dimension has been agreed upon, resulting in performance‐approach goals (striving to perform better than others to demonstrate competence) and performance‐avoidance goals (striving to avoid performing worse than others to not demonstrate incompetence; Elliot, 1999; Janke et al., 2016; Murayama et al., 2011).

Achievement goals have been conceptualized and empirically tested as relatively stable, trait‐like orientations, but they can also be prompted situationally in achievement settings such as classrooms and change over the course of the school years (Ames & Archer, 1988; Linnenbrink, 2005). Achievement goals are assumed to be partly independent from each other; that is, individuals can simultaneously pursue more than one goal (Nicholls, 1984). Predictors of achievement goals are individual factors, including needs, beliefs, or self‐perception (Elliot & Church, 1997), but also the learning context (see Butera et al., 2024 for a recent review). Social norms as a contextual factor have not been explicitly addressed in this research yet. However, we argue that inferences about the role of injunctive norms for individual achievement goals can be drawn from research on classroom goal structures.

### Classroom goal structures as injunctive norms

Students in classrooms emphasizing specific achievement goals are more likely to adopt and emphasize the same goals (Meece et al., 2006). This formation of achievement goals within the school environment has been studied within the framework of classroom goal structures. Classroom goal structures refer to which achievement goals are reinforced and made salient within a context (e.g., by the teacher; Ames, 1992; Midgley et al., 2000; Urdan & Schoenfelder, 2006). Those salient goal structures influence student motivation (i.e., achievement goals) and learning behaviour such as effort, help‐seeking, or self‐handicapping (Lau & Nie, 2008; Meece et al., 2006; Ryan & Patrick, 2001; Urdan et al., 1998). Teachers convey classroom goal structures through their use of instructional practices including evaluation, grouping, or task assignment (Ames, 1992; Lüftenegger et al., 2017). As such, classroom goal structures essentially reflect injunctive norms as they convey messages about what behaviour is accepted and are distributed through social interaction.

Although classroom goal structures are conceptualized as characteristics of the learning context, theoretically, no conceptual consensus is apparent on the operational level. Many researchers rely on individual‐level perceptual measures (e.g., Karabenick, 2004; Lau & Nie, 2008; Murayama & Elliot, 2009; Ryan et al., 1998; Wolters, 2004). This choice reflects a view of classroom goal structures as an individually construed reality (i.e., a ‘psychological environment’; Maehr & Midgley, 1991 or ‘microclimate’; Robinson, 2023). For the adoption of achievement goals, relations with such *individual‐level classroom goal structures* have been shown in several studies, with some using longitudinal designs (e.g., Bong, 2005; Greene et al., 2004; Skaalvik & Federici, 2016; Urdan, 2004).

While this research is valuable, we argue that it does not effectively reflect the learning context. Operationalizations of classroom goal structures from individual student perceptions (level1) can reflect aspects of the shared context effectively. However, this context is only represented empirically if these ratings are aggregated to the group level, that is, the classroom (Lüdtke et al., 2009; Marsh et al., 2012). The role of such *class‐level classroom goal structures* (i.e., ‘climate’; Robinson, 2023) for individual achievement goals is comparatively less understood and is limited to cross‐sectional data. Mastery goal structures relate consistently to individual mastery goals (e.g., Baudoin & Galand, 2020; Murayama & Elliot, 2009). For performance‐approach and performance‐avoidance goal structures, results are mixed. Some studies found positive relations of performance‐approach goal structures with individual performance‐approach goals and performance‐avoidance goal structures with individual performance‐avoidance goals (e.g., Luo et al., 2011); others found no significant relations (see Bardach et al., 2020; Baudoin & Galand, 2020; Murayama & Elliot, 2009). To address our research question and extend prior studies, we test the longitudinal relations of class‐level classroom goal structures representing injunctive norms to changes in individual achievement goals.

### Peer achievement goals as descriptive norms

Although not addressing injunctive norms explicitly, research on classroom goal structures provides some indications about their role in shaping individual achievement goals. Similarly, descriptive norms in the classroom have not been addressed explicitly yet. We think that peer achievement goals can act as descriptive norms. In the classroom, classmates represent a crucial peer context. From childhood to adolescence, students spend a considerable amount of time in school with other classmates or friends, making the peer context especially salient (Brown, 2004; Rodkin & Ryan, 2012). Particularly during adolescence, peers influence values, attitudes, behaviour, and motivation, and are the most important source of normative influence (Brown, 2004; Ladd et al., 2009; Ryan, 2003; Steinberg & Monahan, 2007; Wentzel et al., 2004). By aligning their own attitudes and behaviour to peers' attitudes and behaviour, adolescents detach successfully from parental values, which is an important part of identity formation (Brown, 1990; Harter et al., 1996). From an identity‐based theoretical perspective, emulating peer behaviour can be seen as a means of fostering a positive self‐image by conforming to descriptive norms that define accepted behaviour (see Brown, 2004). Although peers are often operationalized as close friends, researchers in the educational context also rely on the entire class. In most school systems, students remain in fixed groups of classmates across subjects and over significant periods of their education. Thus, all classmates can be frequent interaction partners and be referred to as peers.

Peers influence student motivation and achievement. Previous research has shown relations to autonomous motivation, changes in individual enjoyment, boredom, or error reactions (Reindl et al., 2015, 2018; Ryan, 2003; Tulis et al., 2018). *Perceived* peer achievement goals as determinants of individual achievement goals have been addressed mainly using cross‐sectional designs. For example, Jiang et al. (2014) tested relations between perceived peer and teacher achievement goals and individual motivation in mathematics. Perceived peer achievement goals predicted individual achievement goals more strongly than perceived teacher achievement goals. Hemi et al. (2023a) replicated and extended these results. They found that perceived peer achievement goals explained more variance in individual achievement goals than perceived teacher achievement goals, and perceived peer achievement goals mediated associations between perceived teacher achievement goals and individual achievement goals. Recently, evidence from longitudinal data additionally suggests that perceived teacher and peer achievement goals differentially affect changes in individual achievement goals. Perceived teacher goals predicted individual mastery goals, while perceived peer goals predicted individual performance goals (Hemi et al., 2023b).

These studies, however, only took individually perceived peer achievement goals into account. We think that individual perceptions do not unambiguously reflect the context. Questions remain regarding peer achievement goals as a contextual influence on individual achievement goals. A venture in this direction is a cross‐sectional study by Hemi et al. (2024). Perceived and actual (self‐reported) peer achievement goals (differentiated further into classmates' and social peer groups' goals) were used to predict individual achievement goals using multilevel analyses. Overall, significant relations between peer achievement goals and individual achievement goals emerged. Perceived classmates' and peer achievement goals explained additional variance in individual achievement goals. Taken together, these results suggest that both actual and perceived peer achievement goals are meaningfully related to individual achievement goals. Using a longitudinal approach, King and Mendoza (2020) examined the role of peer achievement goals for individual achievement goals. Mean mastery‐, performance‐approach, and performance‐avoidance goals of the class predicted changes in the corresponding individual achievement goal seven months later. The authors explained the pattern with goal contagion. However, this explanation does not illustrate the reason behind the automatic adoption and pursuit of goals inferred from others (Aarts & Dijksterhuis, 2000), nor does it take social norms into account.

We argue that classmates constitute one of the students' peer contexts and that peer achievement goals act as descriptive norms. We want to investigate the relations between such descriptive norms and individual achievement goals. We expect to replicate previous findings while offering a broader perspective on the underlying processes including classroom goal structures.

### Research question

In sum, we theoretically integrate contextual influences on achievement goals and social norms. We propose that social norms can be applied to explain the influence of classroom goal structures and peer achievement goals on individual achievement goals. By providing a single, broader framework, we aim to enhance our understanding of the combined effect of social contextual influences on individual achievement goals, offering a novel theoretical and methodological approach. We state that social norms drive the influence of classroom goal structures and peer achievement goals on individual achievement goals. We conceptualize classroom goal structures as injunctive norms and peer achievement goals as descriptive norms and expect both to influence individual achievement goals independently (Reno et al., 1993). Such an approach is valuable to identify and connect previously isolated strands of research on contextual influences on individual achievement goals in one theoretical background and enhances our understanding of the individual and combined effects of those contextual factors.

**FIGURE 1:**
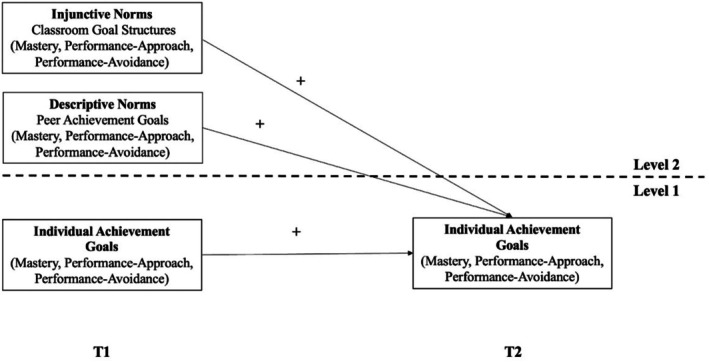
Conceptual model of the relationships between social norms and changes in individual achievement goals. The figure is intended as a conceptual illustration of the theoretical model. For clarity, we refrain from illustrating paths from Level 2 predictors to shared changes from T1 to T2 at Level 2.

Importantly, we think that students are not equally vulnerable to normative influence over the course of their school years. As social norms are especially powerful in ambiguous situations (Lapinski & Rimal, 2005), times of change, such as the transition from primary to secondary education, should make social norms powerful as students try to navigate a new social environment. Additionally, as social norms are formed through interaction, we think that it takes some time for their effects to unfold, which calls for longitudinal data to test our propositions. Our conceptual model is depicted in Figure 1. We propose that (1) injunctive norms represented by class‐level classroom goal structures are related to changes in individual achievement goals over time and that (2) descriptive norms represented by peer achievement goals are related to changes in individual achievement goals over time.

## METHOD

Data from two time points were used for this study, with the first collected four months into 5th grade (T1) and the second two months later (T2). The data were collected from German academic secondary schools (Gymnasium) in the subject of mathematics as part of a larger, two‐year longitudinal project (see Dickhäuser et al., 2017)^1^ that assessed students' individual achievement goals and perceived classroom goal structures across multiple time points. These two time points were selected because, by four months into 5th grade, students have gained some understanding of the prevailing norms in their new academic environment without having fully assimilated, making changes due to normative influence likely.

The final sample included 4198 students from 169 classrooms (one class was excluded because only two individual datasets were available). Half of the sample were girls (*N* = 2099) and boys (*N* = 2094), respectively, with five individuals identifying as neither male nor female. An average of 24.84 students provided data in each class, with a minimum of 13 and a maximum of 32 students.

### Measures

All items were measured on a 6‐point Likert Scale from 1 (*strongly disagree*) to 6 (*strongly agree*). Identifiers for classes included in the data enabled clustering of participants into their respective contexts. Sample items and reliabilities are depicted in Table 1.

**TABLE 1:** Descriptive statistics of relevant variables.

	T1							T2				
	Cronbach's *α*	*N*	*M*	*SD*	ICC1	ICC2	Rwgj	Cronbach's *α*	*N*	*M*	*SD*	ICC1
Achievement goals												
Mastery goals	0.84	3757	4.89	0.74	0.03	0.41	0.88	0.84	3549	4.91	0.84	0.04
*In math, I want to learn something interesting*												
Performance‐approach goals	0.86	3757	3.61	1.01	0.03	0.42	0.79	0.85	3549	3.56	1.05	0.03
*In math, I want to show that I am good at something*												
Performance‐avoidance goals	0.89	3757	2.83	1.09	0.03	0.37	0.72	0.91	3548	2.72	1.16	0.03
*In math, I don't want that others think I am not smart*												
Classroom goal structures												
Mastery goals	0.63	3756	5.09	0.6	0.01	0.18	0.92					
*In our math class, learning new ideas and concepts is very important*												
Performance‐approach goals	0.66	3743	3.76	1.02	0.05	0.55	0.68					
*In our math class, getting right answers is very important*												
Performance‐avoidance goals	0.82	3754	2.63	1.04	0.04	0.47	0.72					
*In our math class, showing others that you are not bad at class work is really important*												

*Note*: Example items in italics.

### Individual achievement goals and peer achievement goals

*Individual achievement goals* were measured at both time points using an established German Achievement Goals Measure (Spinath et al., 2002) adapted for math class. *Peer achievement goals* were calculated as the means of the class‐wise aggregated individual achievement goals (see Marsh et al., 2012).^2^

### Individual‐level and class‐level classroom goal structures

*Individual‐level classroom goal structures* were measured at T1 using a German version of the patterns of adaptive learning scale (PALS; Midgley et al., 2000 adapted by Dresel et al., 2010) adapted for math class. *Class‐level classroom goal structures* were calculated as the means of the classroom's aggregated perceived classroom goal structures.

### Analyses

To test our hypotheses, we calculated three analogous series of analyses to predict each outcome (i.e. individual achievement goal at T2) separately. To get a full picture of the relations between the different constructs, we decided to apply a stepwise procedure. Although this is not necessary to test our overall hypothesis, we see this as a valuable method to disentangle the individual and combined effects of our predictors and understand how the inclusion of both alters relations.

We calculated four multilevel models (M1–M4) to predict each individual achievement goal at T2. In each model, fixed slopes were specified for all predictors, assuming consistent relationships across classes, while allowing for varying intercepts. Model 1 included only individual‐level predictors: the respective achievement goal at T1 (to control for stability), the corresponding individual‐level perceived classroom goal structure, and the other achievement goals as controls (e.g., Fryer & Elliot, 2007; King & Mendoza, 2020). In Models 2 and 3, each of the two class‐level predictors was added individually: Model 2 included the mean classroom goal structure corresponding to the outcome, and Model 3 included the peer achievement goal corresponding to the outcome. Finally, in Model 4, both class‐level predictors were included simultaneously to predict the corresponding individual achievement goal at T2. Models 2 and 3 allowed testing the isolated effects of injunctive and descriptive norms, while Model 4 allowed testing the joint effect of both norms on changes in individual achievement goals.

The overall analytic strategy was analogous to King and Mendoza (2020). Predictors at the individual level were group‐mean‐centered, and predictors at the classroom level were grand‐mean‐centered, as recommended when context effects are analysed using student ratings (see Lüdtke et al., 2009).

## RESULTS

Data were analysed using the R software (R Core Team, 2023), with primary hypotheses tested as multilevel models using the ‘lme4’ and ‘lmeTest’ packages (Bates et al., 2015; Kuznetsova et al., 2017).

Descriptive statistics for all relevant variables are depicted in Table 1 (correlations and intercorrelations see Appendix). Intra‐class coefficients (ICC1) indicate the proportion of between‐classroom variance to total variance. In our sample, all measured constructs showed statistically significant variation between classrooms (all 95% CIs excluding zero). Between 3% and 4% of each individual achievement goal at T2 was explained by variations on the classroom level.

To assess the reliability of aggregate measures, it is common to use the ICC2 (see Lüdtke et al., 2009), which is notably low in our data. However, as can easily be deduced from the formula to compute ICC2 (see Bliese, 1998), a low ICC2 score can result not only from low reliability but also from low variability between classes, which is the case in our sample (see also Bliese, 2000; Marsh et al., 2012). Therefore, we decided to instead inspect rWGj as an indicator of the within‐group interrater agreement. It indicates whether aggregation of data to a higher level is appropriate and is not based on variability between groups (James et al., 1993). The mean rWGj values for our predictors indicate moderate to very strong agreement (LeBreton & Senter, 2008) pointing to the appropriateness of the aggregate measure (see Table 1).

Results of models predicting changes in mastery goals, performance‐approach goals, and performance‐avoidance goals are depicted in Tables 2, 3, 4, respectively. Individual‐level mastery goals and individual‐level classroom mastery goal structures at T1 predicted changes in individual mastery goals at T2. Added to separate models each, class‐level classroom mastery goal structures and peer mastery goals on Level 2 predicted changes in individual mastery goals (M2 and M3). However, if both predictors were added simultaneously, peer mastery goals remained the single significant predictor of changes in individual mastery goals on the class‐level. Results of analogous models predicting performance‐approach and performance‐avoidance goals, respectively, matched this pattern. Individual‐level performance‐approach goals and individual‐level classroom performance‐approach goal structures at T1 predicted changes in individual performance‐approach goals at T2. Added to separate models each, class‐level classroom performance‐approach goal structures and peer performance‐approach goals on Level 2 predicted changes in individual performance‐approach goals (M2 and M3). However, if both predictors were added simultaneously, peer performance‐approach goals remained the single significant predictor of changes in individual performance‐approach goals on the class‐level. Individual‐level performance‐avoidance goals and individual‐level classroom performance‐avoidance goal structures at T1 predicted changes in individual performance‐avoidance goals at T2. Added to separate models each, class‐level classroom performance‐avoidance goal structures and peer performance‐avoidance goals on Level 2 predicted changes in individual performance‐avoidance goals (M2 and M3). However, if both Level 2 predictors were added simultaneously, peer performance‐avoidance goals remained the single most significant predictor of changes in individual performance‐avoidance goals on the class‐level.

**TABLE 2:** Results of multilevel analyses for mastery goals.

	Model 1		Model 2		Model 3		Model 4	
	*b*	*SE*	*b*	*SE*	*b*	*SE*	*b*	*SE*
Individual‐level variables								
Mastery goals	0.55***	0.02	0.55***	0.02	0.55***	0.02	0.55***	0.02
Performance‐approach goals	0.06***	0.02	0.06***	0.02	0.06**	0.02	0.06***	0.02
Performance‐avoidance goals	−0.09***	0.02	−0.09***	0.02	−0.09***	0.02	−0.09***	0.02
Mastery goal structures	0.15***	0.02	0.15***	0.02	0.15***	0.02	0.15***	0.02
Class‐level classroom goal structures								
Mastery			0.25*	0.13			−0.14	0.10
Class‐level achievement goals								
Mastery					0.77***	0.06	0.81***	0.07
Marginal *R* ^2^/conditional *R* ^2^	0.30/0.34		0.30/0.34		0.34/0.34		0.34/0.34	

*Note*: Unstandardized parameters are shown. Marginal *R* ^2^ represents the variance explained by fixed effects; conditional *R* ^2^ represents the variance explained by the entire model.

* *p* < .05.

** *p* < .01.

*** *p* < .001.

**TABLE 3:** Results of multilevel analyses for performance‐approach goals.

	Model 1		Model 2		Model 3		Model 4	
	*b*	*SE*	*b*	*SE*	*b*	*SE*	*b*	*SE*
Individual‐level controls								
Mastery goals	−0.01	0.02	−0.01	0.02	−0.01	0.02	−0.01	0.02
Performance‐approach goals	0.58***	0.02	0.58***	0.02	0.58***	0.02	0.58***	0.02
Performance‐avoidance goals	0.09***	0.02	0.09***	0.02	0.09***	0.02	0.09***	0.02
Performance‐approach goal structures	0.09***	0.02	0.09***	0.02	0.09***	0.02	0.09***	0.02
Class‐level classroom goal structures								
Performance‐approach			0.29***	0.06			−0.02	0.06
Class‐level achievement goals								
Performance‐approach					0.68***	0.06	0.69***	0.06
Marginal *R* ^2^/conditional *R* ^2^	0.41/0.46		0.42/0.46		0.45/0.45		0.45/0.45	

*Note*: Unstandardized parameters are shown. Marginal *R* ^2^ represents the variance explained by fixed effects; conditional *R* ^2^ represents the variance explained by the entire model.

*** *p* < .001.

**TABLE 4:** Results of multilevel analyses for performance‐avoidance goals.

	Model 1		Model 2		Model 3		Model 4	
	*b*	*SE*	*b*	*SE*	*b*	*SE*	*b*	*SE*
Individual‐level controls								
Mastery goals	−0.13***	0.02	−0.13***	0.02	−0.13***	0.02	−0.13***	0.02
Performance‐approach goals	0.15**	0.02	0.15**	0.02	0.15***	0.02	0.15***	0.02
Performance‐avoidance goals	0.48**	0.02	0.48**	0.02	0.48***	0.02	0.48**	0.02
Performance‐avoidance goal structures	0.13***	0.02	0.13***	0.02	0.13***	0.02	0.13***	0.02
Class‐level classroom goal structures								
Performance‐avoidance			0.56***	0.07			0.15	0.10
Class‐level achievement goals								
Performance‐avoidance					0.72***	0.07	0.61***	0.10
Marginal *R* ^2^/conditional *R* ^2^	0.37/0.42		0.39/0.42		0.40/0.42		0.40/0.42	

*Note*: Unstandardized parameters are shown. Marginal *R* ^2^ represents the variance explained by fixed effects; conditional *R* ^2^ represents the variance explained by the entire model.

** *p* < .01.

*** *p* < .001.

## DISCUSSION

To understand individual achievement motivation, considering the context in which learning occurs is essential. We investigated relations between two important social factors located in a student's context—class‐level classroom goal structures and peer achievement goals—and individual mastery, performance‐approach, and performance‐avoidance goals. We employed a novel approach by theoretically integrating them into a social norms framework, proposing that they reflect injunctive and descriptive norms, and investigating their combined effects. We stressed the importance of operationalizing classroom goal structures and peer achievement goals as aggregate class‐level measures to truly reflect the context and used longitudinal data to test our propositions in multilevel models. We found that class‐level classroom goal structures relate to changes in the corresponding individual achievement goals if peer achievement goals are not considered. However, when peer achievement goals are included, relations between class‐level classroom goal structures and changes in individual achievement goals are no longer evident, and peer achievement goals remain the sole predictor on a contextual level. The pattern showed consistently for prediction of changes in mastery, performance‐approach, and performance‐avoidance goals.

### Theoretical and practical implications

We studied relations between classroom goal structures and changes in individual achievement goals. Previous research often overlooked the importance of modelling classroom goal structures on the group level to thoroughly address contextual influences on individual student motivation. Aggregated measures were only used in cross‐sectional designs, and results were inconsistent (e.g., Bong, 2005; Midgley & Urdan, 1995; Urdan, 2004). We add to this literature in two ways. First, we used longitudinal data to investigate relations of class‐level classroom goal structures to changes in individual achievement goals and addressed them as contextual factors while going beyond cross‐sectional designs. Second, following a thorough theoretical integration, we included peer achievement goals as a contextual factor, recognizing the significant role of peers in the classroom through descriptive norms. We found that classroom goal structures as contextual factors are not of primary importance if peers´ achievement goals are considered. Based on these results, one could argue that classroom goal structures may be more influential as individual‐level ‘psychological environment’ (Maehr & Midgley, 1991) than on a contextual level, where peer achievement goals seem to be especially relevant. These findings highlight the importance of a comprehensive theoretical integration of different contextual factors to understand individual and combined effects on individual student outcomes.

Under the assumption that classroom goal structures and peer achievement goals reflect injunctive and descriptive norms, our results indicate that descriptive norms relate to individual achievement goals and do so more than injunctive norms. This is only partly consistent with our propositions since we reasoned that both norms influence individual achievement goals independently. We cannot at this point draw conclusions as to why this is the case. According to the literature, descriptive norms serve as a shortcut to decide what behaviour is most effective (see Smith & Louis, 2008). It is reasonable to assume that adolescents use the behaviour of their peers to navigate the uncertain school environment. Additionally, descriptive norms by peers could be more easily accessible than injunctive norms in the classroom and thus more influential due to their higher salience (Reno et al., 1993), making them especially important in the classroom context. Since this is a first venture into the incorporation of social norms to predict individual achievement motivation, we refrain from drawing conclusions about the general role of injunctive norms for individual achievement goals. However, we see the results of this study as a valuable starting point for further consideration of contextual effects on individual achievement goals in the light of social norms.

The proposed mechanism of social norms influencing individual achievement goals can also complement prior literature showing peer group effects on individual motivation under the framework of goal contagion, which ‘describes how people appear to catch and pursue the goals implied by the behaviors of others in the social environment’ (Laurin, 2016, p. 669). Such ‘spill‐over effects’ have been demonstrated for social goals and achievement goals and are claimed to be the result of an automatic activation of the respective goal if an individual is faced with others who pursue the same goal (Aarts & Dijksterhuis, 2000; King & Mendoza, 2020). However, no clear process is evident for those ‘spill‐over effects’. We think that our social norms approach can be integrated into this framework, which, surprisingly, does not appear to take social norms as a possible overarching theme into account.

The findings can also be put to practical use in learning and teaching. An implication can be that teachers might use social norms to support the adoption of mastery goals, for example, by acknowledging explicitly if one or several students in class express mastery goals, thus making those desirable peer norms salient.

### Limitations and future research

There are several limitations of our research. First, we used mean classroom achievement goals in our analyses to address relations between peer achievement goals and changes in individual achievement goals. While this is a reasonable approach, given our data, we were not able to differentiate peers according to how close they were to each other. However, closer individuals are likely more influential than distal classmates, and norm conformity tends to increase with proximity (Bukowski et al., 2009; Wang & Liao, 2023). We might not have gotten the full picture and possibly underestimated the effect. Future research could address this question by disentangling peer and classroom influences on individual achievement goals.

A further limitation lies in the different referents of injunctive versus descriptive norms. Although not explicitly stated (‘In our math class…’), it is likely that injunctive norms were interpreted as teacher‐driven, while descriptive norms were operationalized with peers as the referent. The strength of normative influence depends on the salience of the norm and the proximity and perceived similarity to the influencing group or individual (e.g., Cialdini et al., 1990; Dimant, 2019; Wang & Liao, 2023). Since peers are likely perceived as more similar and proximal than teachers, and more time is being spent with the class relative to a single subject teacher, it is possible that these factors, in addition to the type of norm, are responsible for differences in effects and limit the generalizability of our results concerning the importance of the norms relative to each other. Our data only captured the constructs for math classes, which mitigates the potential increased salience of peer descriptive norms that results from spending more time with them throughout the school day compared to subject teachers. Future research could address this question, for example, by explicitly separating peer injunctive norms from teacher injunctive norms.

Expanding the previous point, there seems to be more to the impact of social norms on achievement goals than a simple additive effect. To understand the relations, several points need to be considered: First, we need to address the problem of whether injunctive and descriptive norms always align (which we do not assume to be the case), and how mismatching norms impact individual student outcomes. Second, we think that an important missing part of the puzzle lies in how injunctive norms in the classroom might be further specified into injunctive norms originating from the teacher versus injunctive norms originating from peers. We acknowledge that further research is needed to better understand how injunctive and descriptive norms unfold their combined effect in the classroom. Studies including multiple measures at a higher frequency or including the perspectives of teachers might deepen our understanding.

The participants in the current study were enrolled in German schools with a Western background, within an individualistic culture. In individualistic cultures, independence and autonomy are considered central developmental milestones in adolescence (Steinberg, 1990). In contrast, in collectivistic cultures, independence among adolescents is less encouraged (Chen et al., 2018). Future research could benefit from exploring possible differences in observed relations between Western and non‐Western cultures.

Our data set contained very little variation in individual achievement goals between classes. Although we found considerable relations between group‐level variables and changes in individual achievement goals, the overall importance of those variables is comparably limited. We cannot say if the small between‐classroom variation is a characteristic of our sample or a general tendency. Previous research reported different variations ranging from 1% to between 5% and 35% (King & Mendoza, 2020; Meece et al., 2006). Future research could further investigate the magnitude of between‐class variations in individual achievement goals and possible altering factors such as age or cultural background. It is also likely that circumstances in the classroom, such as how long peers have been learning together or whether the subject teacher is also the classroom teacher, alter these between‐classroom variations. Importantly, we do not want to convey the message that norms are the only and major contextual influence on individual achievement goals or that norms about achievement goals are the only norms present in the classroom. However, we want to stress the importance of acknowledging the classroom as a social space and including social norms on motivation in the range of aspects that shape student motivation.

## CONCLUSION

The classroom as part of the learning context shapes individual achievement motivation. We acknowledged its role as a social space and proposed relations of injunctive and descriptive norms, reflected as classroom goal structures and peer achievement goals, with changes in individual achievement goals. We found that peer achievement goals related to changes in individual achievement goals over and above classroom goal structures. The findings stress the importance of considering peers to better understand individual student motivation and are a valuable starting point for integrating social norms into theory and research on contextual effects on individual student motivation.

## AUTHOR CONTRIBUTIONS

**Sophie Bossert:** Conceptualization; writing – original draft; methodology; writing – review and editing; visualization; formal analysis. **Martin Daumiller:** Methodology; writing – review and editing; writing – original draft; supervision; data curation. **Stefan Janke:** Writing – original draft; writing – review and editing; data curation; supervision. **Markus Dresel:** Data curation; writing – review and editing; writing – original draft. **Oliver Dickhäuser:** Writing – original draft; writing – review and editing; supervision; data curation; methodology; conceptualization.

## CONFLICT OF INTEREST STATEMENT

We have no conflicts of interest to disclose.

## Supporting information


Appendix A.

## Data Availability

Data available on request from the authors.

## Footnotes

^1^
Subsamples of this dataset have previously been used to examine different research questions on student motivation and teacher evaluation (Bonefeld et al., 2017; Dickhäuser et al., 2017; Janke et al., 2016; Praetorius et al., 2017). One study also investigated relations between changes in perceived classroom goal structures and changes in individual achievement goals longitudinally (Janke et al., 2022). However, no classroom aggregate measures were included.

^2^
We decided to use all individual student data to calculate overall means for each class (level 2). Accordingly, the individual values of each student are included on the individual and on the class level. We chose to prioritize the ability to run multilevel analyses over correcting for individual‐level values. To illustrate the extent to which the used class‐level values differ from corrected values, we calculated correlations. All correlations were *r* = .99 indicating a negligible bias.

## References

[bjep12756-bib-0001] Aarts, H. , & Dijksterhuis, A. (2000). Habits as knowledge structures: Automaticity in goal‐directed behavior. Journal of Personality and Social Psychology, 78(1), 53–63. 10.1037/0022-3514.78.1.53 10653505

[bjep12756-bib-0002] Ames, C. (1992). Classrooms: Goals, structures, and student motivation. Journal of Educational Psychology, 84(3), 261–271. 10.1037/0022-0663.84.3.261

[bjep12756-bib-0003] Ames, C. , & Archer, J. (1988). Achievement goals in the classroom: Students' learning strategies and motivation processes. Journal of Educational Psychology, 80(3), 260–267. 10.1037/0022-0663.80.3.260

[bjep12756-bib-0004] Bardach, L. , Oczlon, S. , Pietschnig, J. , & Lüftenegger, M. (2020). Has achievement goal theory been right? A meta‐analysis of the relation between goal structures and personal achievement goals. Journal of Educational Psychology, 112(6), 1197–1220. 10.1037/edu0000419

[bjep12756-bib-0005] Bates, D. , Mächler, M. , Bolker, B. , & Walker, S. (2015). Fitting linear mixed‐effects models using lme4. Journal of Statistical Software, 67(1), 1–48. 10.18637/jss.v067.i01

[bjep12756-bib-0006] Baudoin, N. , & Galand, B. (2020). Do achievement goals mediate the relationship between classroom goal structures and student emotions at school? International Journal of School & Educational Psychology, 10(1), 77–93. 10.1080/21683603.2020.1813227

[bjep12756-bib-0007] Bliese, P. D. (1998). Group size, ICC values, and group‐level correlations: A simulation. Organizational Research Methods, 1(4), 355–373.

[bjep12756-bib-0008] Bliese, P. D. (2000). Within group agreement, non‐independence and reliability: Implications for data and analysis. In K. J. Klein & S. W. J. Kozlowski (Eds.), Multilevel theory, research and methods in organizations: Foundations, extensions, and new directions (pp. 349–381). Jossey‐Bass/Wiley.

[bjep12756-bib-0009] Bonefeld, M. , Dickhäuser, O. , Janke, S. , Praetorius, A. K. , & Dresel, M. (2017). Migrationsbedingte Disparitäten in der Notenvergabe nach dem Übergang auf das Gymnasium [Students' grading according to migration background]. Zeitschrift für Entwicklungspsychologie Und Pädagogische Psychologie, 49, 11–23. 10.1026/0049-8637/a000163

[bjep12756-bib-0010] Bong, M. (2005). Within‐grade changes in Korean girls' motivation and perceptions of the learning environment across domains and achievement levels. Journal of Educational Psychology, 97(4), 656–672. 10.1037/0022-0663.97.4.656

[bjep12756-bib-0011] Brown, B. B. (1990). Peer groups and peer cultures. In S. S. Feldman & G. R. Elliott (Eds.), At the threshold: The developing adolescent (pp. 171–196). Harvard University Press.

[bjep12756-bib-0012] Brown, B. B. (2004). Adolescents' relationships with peers. In R. M. Lerner & L. Steinberg (Eds.), Handbook of adolescent psychology (pp. 363–394). 10.1002/9780471726746.ch12

[bjep12756-bib-0013] Bukowski, W. M. , Motzoi, C. , & Meyer, F. (2009). Friendship as process, function, and outcome. In K. H. Rubin , W. M. Bukowski , & B. Laursen (Eds.), Handbook of peer interactions, relationships, and groups (pp. 217–231). Guilford.

[bjep12756-bib-0014] Butera, F. , Dompnier, B. , & Darnon, C. (2024). Achievement goals: A social influence cycle. Annual Review of Psychology, 75, 527–554. 10.1146/annurev-psych-013123-102139 37758239

[bjep12756-bib-0015] Chen, C. C. , Richardson, G. B. , Lai, M. H. C. , Dai, C. L. , & Hays, D. G. (2018). Development and cross‐cultural validity of a brief measure of separation‐individuation. Journal of Child and Family Studies, 27(9), 2797–2810. 10.1007/s10826-018-1174-5

[bjep12756-bib-0016] Cialdini, R. B. , & Goldstein, N. J. (2004). Social influence: Compliance and conformity. Annual Review of Psychology, 55, 591–621. 10.1146/annurev.psych.55.090902.142015 14744228

[bjep12756-bib-0017] Cialdini, R. B. , Kallgren, C. A. , & Reno, R. R. (1991). A focus theory of normative conduct: A theoretical refinement and reevaluation of the role of norms in human behavior. In M. P. Zanna (Ed.), Advances in experimental social psychology (Vol. 24, pp. 201–234). Academic Press.

[bjep12756-bib-0018] Cialdini, R. B. , Reno, R. R. , & Kallgren, C. A. (1990). A focus theory of normative conduct: Recycling the concept of norms to reduce littering in public places. Journal of Personality and Social Psychology, 58(6), 1015–1026. 10.1037/0022-3514.58.6.1015

[bjep12756-bib-0019] Cialdini, R. B. , & Trost, M. R. (1998). Social influence: Social norms, conformity and compliance. In D. T. Gilbert , S. T. Fiske , & G. Lindzey (Eds.), The handbook of social psychology (4th ed., pp. 151–192). McGraw‐Hill.

[bjep12756-bib-0020] Dickhäuser, O. , Janke, S. , Praetorius, A.‐K. , & Dresel, M. (2017). The effects of teachers' reference norm orientations on students' implicit theories and academic self‐concepts. Zeitschrift für Pädagogische Psychologie/German Journal of Educational Psychology, 31, 205–219. 10.1024/1010-0652/a000208

[bjep12756-bib-0021] Dimant, E. (2019). Contagion of pro‐and anti‐social behavior among peers and the role of social proximity. Journal of Economic Psychology, 73, 66–88. 10.1016/j.joep.2019.04.009

[bjep12756-bib-0022] Dresel, M. , Fasching, M. , Steuer, G. , & Berner, V. D. (2010). The role and the interplay of classroom goal structures, individual motivation and general intelligence for (excellent) school achievement in elementary school classrooms. Talent Development and Excellence, 2(1), 63–81.

[bjep12756-bib-0023] Elliot, A. J. (1999). Approach and avoidance motivation and achievement goals. Educational Psychologist, 34(3), 169–189. 10.1207/s15326985ep3403_3

[bjep12756-bib-0024] Elliot, A. J. , & Church, M. A. (1997). A hierarchical model of approach and avoidance achievement motivation. Journal of Personality and Social Psychology, 72(1), 218–232. https://psycnet.apa.org/doi/10.1037/0022‐3514.72.1.218 10.1037//0022-3514.76.4.628 10234849

[bjep12756-bib-0025] Elliot, A. J. , & McGregor, H. A. (2001). A 2 × 2 achievement goal framework. Journal of Personality and Social Psychology, 80(3), 501–519. 10.1037/0022-3514.80.3.501 11300582

[bjep12756-bib-0026] Elliot, A. J. , McGregor, H. A. , & Gable, S. (1999). Achievement goals, study strategies, and exam performance: A mediational analysis. Journal of Educational Psychology, 91(3), 549–563. 10.1037/0022-0663.91.3.549

[bjep12756-bib-0027] Fryer, J. W. , & Elliot, A. J. (2007). Stability and change in achievement goals. Journal of Educational Psychology, 99(4), 700–714. 10.1037/0022-0663.99.4.700

[bjep12756-bib-0028] Greene, B. A. , Miller, R. B. , Crowson, H. M. , Duke, B. L. , & Akey, K. L. (2004). Predicting high school students' cognitive engagement and achievement: Contributions of classroom perceptions and motivation. Contemporary Educational Psychology, 29(4), 462–482. 10.1016/j.cedpsych.2004.01.006

[bjep12756-bib-0029] Harter, S. , Stocker, C. , & Robinson, N. S. (1996). The perceived directionality of the link between approval and self‐worth: The liabilities of a looking gladd self‐orientation among young adolescents. Journal of Research on Adolescence, 6(3), 285–308.

[bjep12756-bib-0030] Hemi, A. , Madjar, N. , Daumiller, M. , & Rich, Y. (2024). Achievement goals of the social peer‐group and the entire class: Relationships with Students' individual achievement goals. Learning and Individual Differences, 115, 102524. 10.1016/j.lindif.2024.102524

[bjep12756-bib-0031] Hemi, A. , Madjar, N. , & Rich, Y. (2023a). Perceived peer and teacher goals: Relationships with students' academic achievement goals. The Journal of Experimental Education, 91(1), 145–165. 10.1080/00220973.2021.1906199

[bjep12756-bib-0032] Hemi, A. , Madjar, N. , & Rich, Y. (2023b). Perceptions of peer and teacher goals predict academic achievement goals among adolescents. The Journal of Experimental Education, 92(4), 692–712. 10.1080/00220973.2023.2229778

[bjep12756-bib-0033] James, L. R. , Demaree, R. G. , & Wolf, G. (1993). Rwg: An assessment of within‐group interrater agreement. Journal of Applied Psychology, 78(2), 306–309. 10.1037/0021-9010.78.2.306

[bjep12756-bib-0034] Janke, S. , Daumiller, M. , Praetorius, A. K. , Dickhäuser, O. , & Dresel, M. (2022). What reduces the adverse development of motivation at the beginning of secondary education: The relationship between student‐perceived teaching practices and changes in students' achievement goals. In R. Lazarides & D. Raufelder (Eds.), Motivation in unterrichtlichen fachbezogenen Lehr‐Lernkontexten: Perspektiven aus Pädagogik, Psychologie und Fachdidaktiken (pp. 179–210). Springer Fachmedien Wiesbaden.

[bjep12756-bib-0035] Janke, S. , Nitsche, S. , Praetorius, A. K. , Benning, K. , Fasching, M. , Dresel, M. , & Dickhäuser, O. (2016). Deconstructing performance goal orientations: The merit of a dimensional approach. Learning and Individual Differences, 50, 133–146. 10.1016/j.lindif.2016.08.013

[bjep12756-bib-0036] Jiang, Y. , Song, J. , Lee, M. , & Bong, M. (2014). Self‐efficacy and achievement goals as motivational links between perceived contexts and achievement. Educational Psychology, 34, 92–117. 10.1080/01443410.2013.863831

[bjep12756-bib-0037] Karabenick, S. A. (2004). Perceived achievement goal structure and college student help seeking. Journal of Educational Psychology, 96(3), 569–581. 10.1037/0022-0663.96.3.569

[bjep12756-bib-0038] King, R. B. , & Mendoza, N. B. (2020). Achievement goal contagion: Mastery and performance goals spread among classmates. Social Psychology of Education, 23(3), 795–814. 10.1007/s11218-020-09559-x

[bjep12756-bib-0039] Koenka, A. C. (2020). Academic motivation theories revisited: An interactive dialog between motivation scholars on recent contributions, underexplored issues, and future directions. Contemporary Educational Psychology, 61, 101831. 10.1016/j.cedpsych.2019.101831

[bjep12756-bib-0040] Kuznetsova, A. , Brockhoff, P. B. , & Christensen, R. H. B. (2017). lmerTest package: Tests in linear mixed effects models. Journal of Statistical Software, 82(13), 1–26. 10.18637/jss.v082.i13

[bjep12756-bib-0041] Ladd, G. W. , Herald‐Brown, S. L. , & Kochel, K. P. (2009). Peers and motivation. In K. R. Wentzel & A. Wigfield (Eds.), Handbook of motivation at school (pp. 323–348). Routledge.

[bjep12756-bib-0042] Lapinski, M. K. , & Rimal, R. N. (2005). An explication of social norms. Communication Theory, 15(2), 127–147. 10.1111/j.1468-2885.2005.tb00329.x

[bjep12756-bib-0043] Lau, S. , & Nie, Y. (2008). Interplay between personal goals and classroom goal structures in predicting student outcomes: A multilevel analysis of person‐context interactions. Journal of Educational Psychology, 100(1), 15–29. 10.1037/0022-0663.100.1.15

[bjep12756-bib-0044] Laurin, K. (2016). Interpersonal influences on goals: Current and future directions for goal contagion research. Social and Personality Psychology Compass, 10(11), 668–678. 10.1111/spc3.12289

[bjep12756-bib-0045] LeBreton, J. M. , & Senter, J. L. (2008). Answers to 20 questions about interrater reliability and interrater agreement. Organizational Research Methods, 11(4), 815–852. 10.1177/1094428106296642

[bjep12756-bib-0046] Legros, S. , & Cislaghi, B. (2020). Mapping the social‐norms literature: An overview of reviews. Perspectives on Psychological Science, 15(1), 62–80. 10.1177/1745691619866455 31697614 PMC6970459

[bjep12756-bib-0047] Linnenbrink, E. A. (2005). The dilemma of performance‐approach goals: The use of multiple goal contexts to promote students' motivation and learning. Journal of Educational Psychology, 97(2), 197–213. 10.1037/0022-0663.97.2.197

[bjep12756-bib-0048] Lüdtke, O. , Robitzsch, A. , Trautwein, U. , & Kunter, M. (2009). Assessing the impact of learning environments: How to use student ratings of classroom or school characteristics in multilevel modeling. Contemporary Educational Psychology, 34(2), 120–131. 10.1016/j.cedpsych.2008.12.001

[bjep12756-bib-0049] Lüftenegger, M. , Tran, U. S. , Bardach, L. , Schober, B. , & Spiel, C. (2017). Measuring a classroom mastery goal structure using the target dimensions: Development and validation of a classroom goal structure scale. Zeitschrift für Psychologie, 225(1), 64–75. 10.1027/2151-2604/a000277

[bjep12756-bib-0050] Luo, W. , Hogan, D. , & Paris, S. G. (2011). Predicting Singapore students' achievement goals in their English study: Self‐construal and classroom goal structure. Learning and Individual Differences, 21(5), 526–535. 10.1016/j.lindif.2011.07.002

[bjep12756-bib-0051] Maehr, M. L. , & Midgley, C. (1991). Enhancing student motivation: A school‐wide approach. Educational Psychologist, 26(3–4), 399–427. 10.1080/00461520.1991.9653140

[bjep12756-bib-0052] Marsh, H. W. , Lüdtke, O. , Nagengast, B. , Trautwein, U. , Morin, A. J. S. , Abduljabbar, A. S. , & Köller, O. (2012). Classroom climate and contextual effects: Conceptual and methodological issues in the evaluation of group‐level effects. Educational Psychologist, 47(2), 106–124. 10.1080/00461520.2012.670488

[bjep12756-bib-0053] Meece, J. L. , Anderman, E. M. , & Anderman, L. H. (2006). Classroom goal structure, student motivation, and academic achievement. Annual Review of Psychology, 57, 487–503. 10.1146/annurev.psych.56.091103.070258 16318604

[bjep12756-bib-0054] Midgley, C. , Maehr, M. L. , Hruda, L. Z. , Anderman, E. , Anderman, L. , Freeman, K. E. , & Urdan, T. (2000). Manual for the patterns of adaptive learning scales (pp. 734–763). University of Michigan.

[bjep12756-bib-0055] Midgley, C. , & Urdan, T. (1995). Predictors of middle school students' use of self‐handicapping strategies. The Journal of Early Adolescence, 15(4), 389–411. 10.1177/0272431695015004001

[bjep12756-bib-0056] Murayama, K. , & Elliot, A. J. (2009). The joint influence of personal achievement goals and classroom goal structures on achievement‐related outcomes. Journal of Educational Psychology, 101(2), 432–447. 10.1037/a0014221

[bjep12756-bib-0057] Murayama, K. , Elliot, A. J. , & Yamagata, S. (2011). Separation of performance‐approach and performance‐avoidance achievement goals: A broader analysis. Journal of Educational Psychology, 103(1), 238–256. 10.1037/a0021948

[bjep12756-bib-0058] Nicholls, J. G. (1984). Achievement motivation: Conceptions of ability, subjective experience, task choice, and performance. Psychological Review, 91(3), 328–346. 10.1037/0033-295X.91.3.328

[bjep12756-bib-0059] Payne, S. C. , Youngcourt, S. S. , & Beaubien, J. M. (2007). A meta‐analytic examination of the goal orientation nomological net. Journal of Applied Psychology, 92(1), 128–150. 10.1037/0021-9010.92.1.128 17227156

[bjep12756-bib-0060] Praetorius, A.‐K. , Lauermann, F. , Klassen, R. M. , Dickhäuser, O. , Janke, S. , & Dresel, M. (2017). Longitudinal relations between teaching‐related motivations and student‐reported teaching quality. Teaching and Teacher Education, 65, 241–254. 10.1016/j.tate.2017.03.023

[bjep12756-bib-0061] R Core Team . (2023). R: A language and environment for statistical computing. R Foundation for Statistical Computing. https://www.R‐project.org/

[bjep12756-bib-0062] Reindl, M. , Berner, V. D. , Scheunpflug, A. , Zeinz, H. , & Dresel, M. (2015). Effect of negative peer climate on the development of autonomous motivation in mathematics. Learning and Individual Differences, 38, 68–75. 10.1016/j.lindif.2015.01.017

[bjep12756-bib-0063] Reindl, M. , Tulis, M. , & Dresel, M. (2018). Associations between friends, academic emotions and achievement: Individual differences in enjoyment and boredom. Learning and Individual Differences, 62, 164–173. 10.1016/j.lindif.2018.01.017

[bjep12756-bib-0064] Reno, R. R. , Cialdini, R. B. , & Kallgren, C. A. (1993). The transsituational influence of social norms. Journal of Personality and Social Psychology, 64(1), 104–112. 10.1037/0022-3514.64.1.104

[bjep12756-bib-0065] Robinson, K. A. (2023). Motivational climate theory: Disentangling definitions and roles of classroom motivational support, climate, and microclimates. Educational Psychologist, 58(2), 92–110. 10.1080/00461520.2023.2198011

[bjep12756-bib-0066] Rodkin, P. C. , & Ryan, A. M. (2012). Child and adolescent peer relations in educational context. In K. R. Harris , S. Graham , T. Urdan , S. Graham , J. M. Royer , & M. Zeidner (Eds.), APA educational psychology handbook, 2. Individual differences and cultural and contextual factors (pp. 363–389). American Psychological Association. 10.1037/13274-015

[bjep12756-bib-0067] Roeser, R. W. , Urdan, T. C. , & Stephens, J. M. (2009). School as a context of student motivation and achievement. In K. R. Wentzel & D. B. Miele (Eds.), Handbook of motivation at school (pp. 395–424). Routledge.

[bjep12756-bib-0068] Ryan, A. M. (2003). The peer group as a context for the development of young adolescent motivation and achievement. Child Development, 72(4), 1135–1150. 10.1111/1467-8624.00338 11480938

[bjep12756-bib-0069] Ryan, A. M. , Gheen, M. H. , & Midgley, C. (1998). Why do some students avoid asking for help? An examination of the interplay among students' academic efficacy, teachers' social–emotional role, and the classroom goal structure. Journal of Educational Psychology, 90(3), 528–535. 10.1037/0022-0663.90.3.528

[bjep12756-bib-0070] Ryan, A. M. , & Patrick, H. (2001). The classroom social environment and changes in adolescents' motivation and engagement during middle school. American Educational Research Journal, 38(2), 437–460. 10.3102/00028312038002437

[bjep12756-bib-0071] Schunk, D. H. , & Mullen, C. A. (2012). Self‐efficacy as an engaged learner. In S. Christenson , A. Reschly , & C. Wylie (Eds.), Handbook of research on student engagement (pp. 219–235). Springer. 10.1007/978-1-4614-2018-7_10

[bjep12756-bib-0072] Senko, C. , & Dawson, B. (2017). Performance‐approach goal effects depend on how they are defined: Meta‐analytic evidence from multiple educational outcomes. Journal of Educational Psychology, 109(4), 574–598. 10.1037/edu0000160

[bjep12756-bib-0073] Sherif, M. (1936). The psychology of social norms. Harper.

[bjep12756-bib-0074] Skaalvik, E. M. , & Federici, R. A. (2016). Relations between classroom goal structures and students' goal orientations in mathematics classes: When is a mastery goal structure adaptive? Social Psychology of Education, 19, 135–150.

[bjep12756-bib-0075] Smith, J. R. , & Louis, W. R. (2008). Do as we say and as we do: The interplay of descriptive and injunctive group norms in the attitude–behaviour relationship. British Journal of Social Psychology, 47(4), 647–666. 10.1348/014466607X269748 18163950

[bjep12756-bib-0076] Spinath, B. , Stiensmeier‐Pelster, J. , Schöne, C. , & Dickhäuser, O. (2002). Skalen zur Erfassung der Lern‐ und Leistungsmotivation: SELLMO. Hogrefe.

[bjep12756-bib-0077] Steinberg, L. (1990). Autonomy, conflict, and harmony in the family relationship. In S. Feldman & G. Elliot (Eds.), At the threshold: The developing adolescent (pp. 255–276). Harvard University Press.

[bjep12756-bib-0078] Steinberg, L. , & Monahan, K. C. (2007). Age differences in resistance to peer influence. Developmental Psychology, 43(6), 1531–1543. 10.1037/0012-1649.43.6.1531 18020830 PMC2779518

[bjep12756-bib-0079] Steinel, W. , Van Kleef, G. A. , Van Knippenberg, D. , Hogg, M. A. , Homan, A. C. , & Moffit, G. (2010). How intragroup dynamics affect behavior in intergroup conflict: The role of group norms, prototypicality, and need to belong. Group Processes & Intergroup Relations, 13(6), 779–794. 10.1177/1368430210375702

[bjep12756-bib-0080] Tulis, M. , Reindl, M. , & Dresel, M. (2018). Freundschaften im Klassenzimmer und deren Bedeutung für einen adaptiven individuellen Umgang mit Fehlern. Zeitschrift für Entwicklungspsychologie Und Pädagogische Psychologie, 50(1), 44–58. 10.1026/0049-8637/a000186

[bjep12756-bib-0081] Urdan, T. (2004). Predictors of academic self‐handicapping and achievement: Examining achievement goals, classroom goal structures, and culture. Journal of Educational Psychology, 96(2), 251–264. 10.1037/0022-0663.96.2.251

[bjep12756-bib-0082] Urdan, T. , Midgley, C. , & Anderman, E. M. (1998). The role of classroom goal structure in students' use of self‐handicapping strategies. American Educational Research Journal, 35(1), 101–122. 10.3102/00028312035001101

[bjep12756-bib-0083] Urdan, T. , & Schoenfelder, E. (2006). Classroom effects on student motivation: Goal structures, social relationships, and competence beliefs. Journal of School Psychology, 44(5), 331–349. 10.1016/j.jsp.2006.04.003

[bjep12756-bib-0084] Urhahne, D. , & Wijnia, L. (2023). Theories of motivation in education: An integrative framework. Educational Psychology Review, 35(2), 45. 10.1007/s10648-023-09767-9

[bjep12756-bib-0085] Wang, E. , & Liao, Y. T. (2023). Effects of member similarity on group norm conformity, group identity and social participation in the context of social networking sites. Internet Research, 34(3), 868–890. 10.1108/INTR-09-2021-0632

[bjep12756-bib-0086] Wentzel, K. R. , Barry, C. M. , & Caldwell, K. A. (2004). Friendships in middle school: Influences on motivation and school adjustment. Journal of Educational Psychology, 96(2), 195–203. 10.1037/0022-0663.96.2.195

[bjep12756-bib-0087] Wolters, C. A. (2004). Advancing achievement goal theory: Using goal structures and goal orientations to predict students' motivation, cognition, and achievement. Journal of Educational Psychology, 96(2), 236–250. 10.1037/0022-0663.96.2.236

