# Supplementary material for: On the influence of social norms on individual achievement goals
Source: Br J Educ Psychol. 2025 Mar 1;95(4):924–40. doi: 10.1111/bjep.12756 (PMC12590932; doi:10.1111/bjep.12756)
Supplement: Supplementary file 1 — Appendix A. [file BJEP-95-924-s001.docx]

| Variable | 1 | 2 | 3 | 4 | 5 | 6 | 7 | 8 | 9 | 10 | 11 | 12 | 13 | 14 | 15 |
| --- | --- | --- | --- | --- | --- | --- | --- | --- | --- | --- | --- | --- | --- | --- | --- |
| **T1**  **Individual Level** |  |  |  |  |  |  |  |  |  |  |  |  |  |  |  |
| 1. Mastery Goals |  |  |  |  |  |  |  |  |  |  |  |  |  |  |  |
| 1. Performance-Approach Goals | .31*** |  |  |  |  |  |  |  |  |  |  |  |  |  |  |
| 1. Performance-Avoidance Goals | .01 | .68*** |  |  |  |  |  |  |  |  |  |  |  |  |  |
| 1. Classroom Mastery Goals | .45*** | .23*** | .06*** |  |  |  |  |  |  |  |  |  |  |  |  |
| 1. Classroom Performance-Approach Goals | .18*** | .45*** | .38*** | .35*** |  |  |  |  |  |  |  |  |  |  |  |
| 1. Classroom Performance-Avoidance Goals   **Class-Level** | .04* | .54*** | .61*** | .16*** | .58*** |  |  |  |  |  |  |  |  |  |  |
| 1. Mastery Goals | .27*** | .08*** | -.02 | .08*** | .03 | -.02 |  |  |  |  |  |  |  |  |  |
| 1. Performance-Approach Goals | .08*** | .27*** | .19*** | .05** | .16*** | .18*** | .31*** |  |  |  |  |  |  |  |  |
| 1. Performance-Avoidance Goals | -.02 | .20*** | .26*** | .02 | .13*** | .21*** | -.03* | .75*** |  |  |  |  |  |  |  |
| 1. Mastery Goal Structure | .09*** | .05*** | .02 | .23*** | .10*** | .04** | .33*** | .21*** | .09*** |  |  |  |  |  |  |
| 1. Performance-Approach Goal Structure | .03 | .14*** | .11*** | .07*** | .30*** | .18*** | .08*** | .51*** | .43*** | .32*** |  |  |  |  |  |
| 1. Performance-Avoidance Goal Structure | -.02 | .17*** | .19*** | 0.03* | .20*** | .28*** | -.06*** | .64*** | .73*** | .16*** | .64*** |  |  |  |  |
| **T2**  **Individual Level** |  |  |  |  |  |  |  |  |  |  |  |  |  |  |  |
| 1. Mastery Goals | .57*** | .17*** | -.06** | .33*** | .13*** | -.00 | .19*** | .04 | -.04 | .05 | .04 | -.03 |  |  |  |
| 1. Performance-Approach Goals | .17*** | .66*** | .50*** | .16*** | .36*** | .44*** | .02 | .18*** | .13*** | .02 | .09*** | .12*** | .21*** |  |  |
| 1. Performance-Avoidance Goals | -.05** | .48*** | .62*** | .03 | .29*** | .46*** | -.05* | .14*** | .17*** | .01 | .10*** | .14*** | -.11*** | .70*** |  |
| *Note.* **p* < .05. ***p* < .01. ****p* < .001. | | | | | | | | | | | | | | | |

**Table A1**

*Correlations and intercorrelations for all relevant variables*
